# Atrial fibrillation evolution and rhythm control strategy following left appendage closure: new insights from the prospective FLAAC registry

**DOI:** 10.1186/s12872-021-01994-8

**Published:** 2021-05-03

**Authors:** Nicolas Lellouche, Raphaele Arrouasse, Julien Ternacle, Romain Gallet, Jean-Sylvain Hermida, David Hamon, Jean-Michel Juliard, Jean-Luc Pasquie, Tarvinder Dhanjal, Emmanuel Teiger, Philippe Le Corvoisier

**Affiliations:** 1 Cardiology Unit Henri Mondor University Hospital Paris XII, Creteil, France; 2 Inserm, CIC 1430, Henri Mondor University Hospital, Creteil, France; 3 Department of Cardiac Arrhythmia, Picardie University Hospital, Amiens, France; 4 Department of Cardiology, Bichat Hospital, APHP, University Paris VII, DHU FIRE, Sorbonne Paris-Cité, INSERM U-1148, Paris, France; 5 Montpellier University Hospital, CNRS UMR9214-Inserm U1046–PHYMEDEXP, Montpellier, France; 6 Department of Electrophysiology, University Hospital Coventry & Warwickshire, Coventry, UK; 7 Inserm, U955 team 3, Henri Mondor University Hospital, Creteil, France; 8 AP-HP, University Hospital Henri Mondor, 51, Avenue du Maréchal de Lattre de Tassigny, 94000 Créteil, France

**Keywords:** Left atrial appendage closure, Atrial fibrillation, Cardioversion, Ablation

## Abstract

**Background:**

Percutaneous left atrial appendage (LAA) closure is an alternative to oral anticoagulation (OAC) for atrial fibrillation (AF) patients with high thromboembolism risk, particularly with contraindications to OAC. The LAA itself could possess proarrhythmogenic properties. As patients undergoing LAA closure could be candidates for cardioversion or ablation, we aimed to evaluate AF disease progression following LAA closure and the outcome of patients undergoing a rhythm control strategy after the procedure.

**Methods:**

The prospective multicenter French Nationwide Observational LAA Closure Registry (FLAAC) comprises 33 French interventional cardiology departments. Patients were included if they fulfilled the following criteria: history of non-valvular AF, successful LAA closure and long-term ECG follow-up.

**Results:**

A total of 331 patients with successful LAA closure were enrolled in the study. Patients mean age was 75.4 ± 0.5 years. The study population was characterized by a high thromboembolic risk (CHA_2_DS_2_-VASc score: 4.5 ± 0.1) and frequent comorbidities. The median follow-up was 11.9 months. One hundred and nineteen (36.0%) patients were in sinus rhythm (SR) at baseline. Among SR patients, documented AF was observed in 16 (13.4%) patients whereas 15 (7.1%) patients in AF at baseline restored SR, at the end of follow up. Finally, only 13 patients (4%) underwent procedures to restore SR without complications during the follow-up.

**Conclusions:**

The vast majority of patients undergoing LAA closure have the same AF status at baseline and one year after the index procedure. During the follow-up, a very small proportion (4%) of our population underwent procedures to restore SR without complications whatever the post-procedural antithrombotic strategy was.

## Background

Atrial fibrillation (AF) is the most common arrhythmia encountered in everyday practice. AF increases the risk of heart failure, stroke and cardiovascular mortality [1–3]. The left atrial appendage (LAA) is the main site for AF-related thrombus formation, responsible for stroke [4, 5]. Over the past decade, a new technique has emerged for AF stroke prevention, namely percutaneous LAA closure. This technic has proven to reduce AF-related stroke and is an alternative to oral anticoagulants (OAC) [6–8], importantly in patients with definitive OAC contraindications [9].

Concomitantly the LAA structure has been shown to have proarrythmogenic properties and has been implicated in the pathophysiology of persistent AF maintenance [10]. Numerous studies have demonstrated that LAA electrical isolation during persistent AF ablation can improve AF ablation outcomes [11–13]. Importantly, the potential effect of LAA closure devices used for LAA closure on cardiac rhythm remains unknown.

Cardioversion and AF catheter ablation are frequently performed to restore and maintain sinus rhythm (SR). These procedures need to be framed by OAC to reduce the risk of stroke [14]. Patients with LAA closure who are contraindicated to OAC could also be candidate for cardioversion and/or catheter ablation. However, there is little known about the long term safety and efficacy within this patient population.

The aim of our study was to evaluate AF burden and AF management including cardioversion and catheter ablation of patients post-LAA closure.

## Methods

### Design of the study

Patients were enrolled in this prospective, observational, multicenter, cohort from April 2013 to September 2015 in 33 French interventional cardiology departments. Center selection was independent of operator experience to ensure that our patients were representative of daily practice.

The study protocol was approved by a national ethics committee and the study was performed in accordance with the ethical principles stated in the Declaration of Helsinki. All patients gave written inform consent prior to the procedure.

We have previously reported the clinical characteristics and outcome of a cohort of 436 patients included in this registry [15]. Inclusion criteria in the current study were the following: (i) history of non-valvular AF, (ii) successful LAA closure and (iii) long-term ECG follow-up (6 or 12 month visits). Exclusion criteria were (i) age under 18 years old and (ii) subjects unable to sign consent form.

This investigator-initiated study was funded by unrestricted grants from the device manufacturers, who had no role in the study design, data collection and interpretation, or writing of the manuscript.

### Left atrial appendage closure and Follow-up

The typical procedure and follow-up were previously described [15]. In summary, a transesophageal echocardiography (TEE) or a cardiac computed tomography angiography (CTA) was performed a few days before LAA closure to assess the size and shape of the LAA and exclude LAA thrombus. The procedure was performed with fluoroscopy and TEE guidance*.* Both Amplatzer Cardiac Plug™/Amulet™ (Abbott Structural Heart, Plymouth, Minnesota, USA) and Watchman*®* devices were commercially available in France during the study period. The choice of antithrombotic treatment prescribed at discharge was based on manufacturer recommendations, patient history and non-cardiovascular comorbidities.

The post procedural management of these patients was left at the discretion of their cardiologist. Patients were instructed to contact their referring cardiologist in case of adverse event or hospitalization during the follow-up period*.* At each visit, clinical symptoms, thromboembolic events, hemorrhagic events, hospitalization, TEE and cardiac CTA-scan results, anti-arrhythmic and antithrombotic treatments were recorded. The cardiac rhythm was assessed during these visits by 12 lead-ECG. Data on interventional procedure (cardioversion, overdrive, catheter ablation) were obtained from patient’s medical records.

### Data management

#### Monitoring

All case report forms were monitored prospectively by an independent research technician*.* Data were entered in an ACCESS database (Microsoft®, Redmond, Washington, USA) and were subjected to quality control procedures. Missing data and outliers were checked against source documents for completeness and accuracy. All clinical adverse events were adjudicated by an independent committee as (i) related or possibly related to the procedure or (ii) not related to the procedure. All thromboembolic events were classified according to the valve Academic Research Consortium (VARC 2) classification [16]. Thromboembolic and bleeding risk were quantified according to the CHA_2_DS_2_-VASc and HAS-BLED scores, respectively.

#### Endpoint

The endpoints of this study were: (i) the rate of AF status modification at the end of the follow up period in patients before LAA closure, (ii) the percentage of patients who underwent electrical cardioversion or catheter ablation and their outcomes during the follow up period.

#### Statistics

Descriptive results are displayed as mean ± SEM or median (interquartile range) for continuous variables, according to the normality of the distributions. Categorical data are described as number (percentage). Categorical variables were compared using the χ^2^ test or Fisher’s exact tests and continuous variables using the Student or Mann–Whitney tests, as appropriate. Two-sided *p* values < 0.05 were considered statistically significant.

## Results

### Study population

A total of 331 patients with successful LAA closure were enrolled in the study. The main characteristics of the population are presented in Table 1. Patients mean age was 75.4 ± 0.5 years and most of the patients were men (n = 215*,* 65.0%). The study population was characterized by a high thromboembolic risk (CHA_2_DS_2_-VASc score: 4.5 ± 0.1) and frequent comorbidities (Table 1). A history of hemorrhagic event was present in 304 (91.8%) patients (HAS-BLED score: 3.1 ± 0.1). Of note, 100 and 231 patients experienced paroxysmal and persistent/permanent AF, respectively.

**Table 1:** Patient baseline characteristics

Patient characteristics	All patients n = 331
Age, years^a^	75.4 ± 0.5
Male Sex, n (%)	215 (65.0)
Risk factors, n (%)	
Hypertension	283 (85.5)
Diabetes mellitus	103 (31.1)
Smoker (current or past)	98 (29.6)
Dyslipidemia	158 (47.7)
Cardiovascular history, n (%)	
Heart failure	91 (27.5)
Coronary artery disease	110 (33.2)
Myocardial infarction	35 (10.6)
Previous deep vein thrombosis/pulmonary embolism	34 (10.3)
Previous ischemic stroke, n (%)	127 (38.4)
Previous hemorrhagic event, n (%)	304 (91.8)
*Hemorrhagic stroke*	100 (30.2)
Subarachnoid hemorrhage	10 (3.0)
Epidural/subdural hematoma	33 (10.0)
Ischemic *stroke with* hemorrhagic *transformation*	19 (5.7)
Gastro-intestinal bleeding	90 (27.2)
*Spontaneous hematoma*	40 (12.1)
Other co-morbidities	
Cirrhosis, n (%)	13 (3.9)
Chronic obstructive pulmonary disease, n (%)	43 (13.0)
Thyroid dysfunction, n (%)	69 (20.8)
Creatinine at admission (µmol/l)^b^	99 [80–129]
Creatinine clearance at admission, mL/min^b^	62 [43–78]
Hemoglobin at admission (g/dl)^b^	13 [11.6–14.2]
CHA_2_DS_2_-VASc score^a^	4.5 ± 0.1
HAS-BLED score^a^	3.1 ± 0.1

^a^Mean ± SEM

^b^Median (interquartile range)

The most common indications for LAA closure were: (i) contraindication to anticoagulation in AF patients with a high thromboembolic risk (95.8%) and (ii) history of thromboembolic event despite an adequate anticoagulation (3.6%). A Watchman® device was implanted in 137 (41.4%) patients, an Amplatzer Cardiac Plug™/Amulet™ in 192 (58.0%) patients and another device in 2 patients (0.6%). The device selected after LAA sizing was adequate in most of the patients, and only 1.08 ± 0.02 devices were used per procedure*.* The median procedural time was 60 [47.5–75] minutes and the mean fluoroscopy time 12 [8.2–18] minutes*.* Procedure related serious adverse events occurred in 19 patients (5.7%), including 2 device embolizations (0.6%) and 6 pericardial effusions requiring pericardiocentesis (1.8%)*.*

All patients enrolled in this study had a prior history of AF diagnosed on average 3.0 [0.7–6.2] years before LAA closure, and 34 (10.3%) of them have previously undergone an ablation procedure. One hundred and nineteen (36.0%) patients were in SR (SR group) at the baseline visit and 212 (64.0%) in AF (AF group). There was no statistically significant difference in baseline characteristics between both groups (Table 2).

**Table 2:** Patient characteristics according to cardiac rhythm at baseline

Patient characteristics	Sinus rhythm at admission n = 119	Atrial fibrillation at admission n = 212	*p*
Age, years^a^	74.8 ± 0.8	75.7 ± 0.6	0.33
Male Sex, n (%)	70 (58.8)	145 (68.4)	0.08
Risk factors, n (%)			
Hypertension	105 (88.2)	178 (84.0)	0.29
Diabetes mellitus	42 (35.3)	61 (28.8)	0.22
Smoker (current or past)	33 (27.7)	65 (30.7)	0.58
Dyslipidemia	61 (51.3)	97 (45.8)	0.34
Cardiovascular history, n (%)			
Heart failure	26 (21.8)	65 (30.7)	0.08
Coronary artery disease	37 (31.1)	73 (34.4)	0.54
Myocardial infarction	13 (10.9)	22 (10.4)	0.88
Previous deep vein thrombosis/ pulmonary embolism	12 (10.1)	22 (10.4)	0.93
Previous ischemic stroke, n (%)	52 (43.7)	75 (35.4)	0.14
Previous hemorrhagic event, n (%)	108 (90.8)	196 (92.5)	0.59
Other co-morbidities			
Cirrhosis, n (%)	2 (1.7)	11 (5.2)	0.15
Chronic obstructive pulmonary disease, n (%)	13 (10.9)	30 (14.2)	0.40
Thyroid dysfunction, n (%)	20 (16.8)	49 (23.1)	0.18
Creatinine at admission (µmol/l)^b^	96 [77–126]	102 [82–130]	0.34
Creatinine clearance at admission, mL/min^b^	61 [46–78]	62 [43–78]	0.71
Hemoglobin at admission (g/dl)^b^	13.2 [11.7–14.3]	12.9 [11.5–14.1]	0.42
CHA_2_DS_2_-VASc score^a^	4.6 ± 0.2	4.5 ± 0.1	0.40
HAS-BLED score^a^	3.1 ± 0.1	3.2 ± 0.1	0.31

^a^Mean ± SEM

^b^Median (interquartile range)

### Procedures

Only few patients (13 patients; 4%) included in the FLAAC registry underwent a specific rhythm control intervention during the follow-up period after LAA closure. The clinical characteristics of these patients and the details of these procedures are presented in Table 4.

Four patients with persistent AF or flutter underwent electrical cardioversion or overdrive pacing via an implanted pacemaker (only for atrial flutter). These procedures were performed on average 6.3 ± 1.9 months after LAA closure and restored SR in all of these patients*.*

**Fig. 1:**
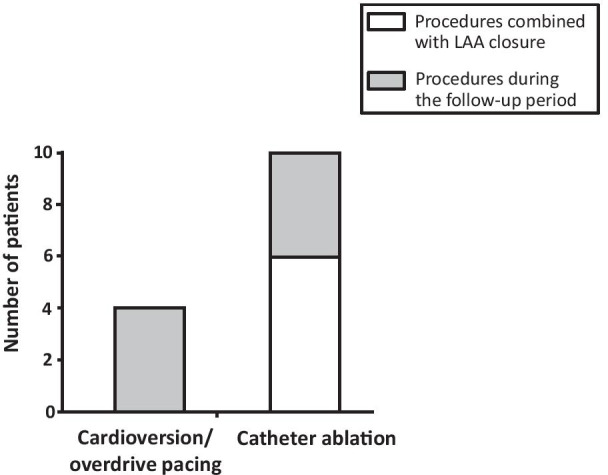
Time between LAA closure and cardioversion or catheter ablation

Ten procedures of catheter ablation of atrial flutter or fibrillation were performed during the follow-up period, in 9 patients. Six of these procedures were combined with LAA closure (Fig. 1). These interventions were a first ablation procedure in 5 patients and a redo procedure in 4. The procedure was successful in all patients and no major adverse event was observed during the follow-up period.

Only 6 of these 13 patients received anticoagulant agents during the days or weeks before hospitalization for catheter ablation or electrical cardioversion. The baseline antithrombotic treatment of the patients was strengthened after the procedure in 5 of them.

Of note, the population that underwent cardioversion or ablation of an AF during the follow up period was characterized by a lower CHA_2_DS_2_-VASc (2.9 ± 0.4 vs. 4.6 ± 0.1, *p* < 0.001) and HAS-BLED scores (2.3 ± 0.3 vs. 3.2 ± 0.1, *p* < 0.01) and was younger (70.5 ± 2.3 vs.75.6 ± 0.5, *p* < 0.05) than the general population included in the registry.

### Follow up

**Fig. 2:**
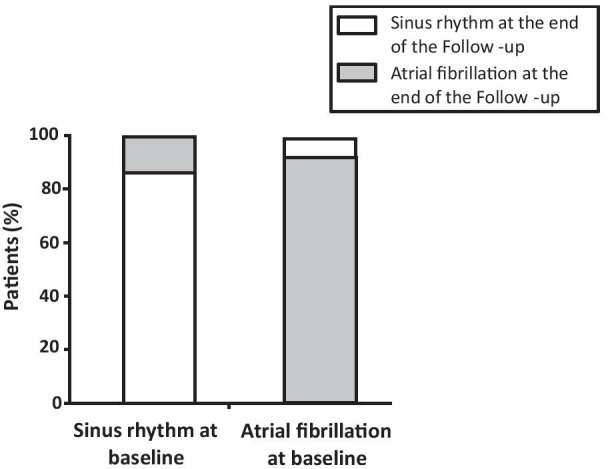
Changes in cardiac rhythm during the follow-up period

Patients were followed up for 11.9 [9.2–13.7] months. Among patients with SR before LAA closure, 103 patients (86.6%) remained in SR at the end of follow up and a documented recurrence of AF was observed in the remaining 16 (13.4%) (Fig. 2). The median time to AF recurrence was 6.1 [1.1–12.4] months after LAA closure.

Similarly, 15 (7.1%) patients with AF at baseline restored SR at the end of the follow up period. Changes in cardiac rhythm after LAA closure are summarized in Fig. 2.

A total of 8 ischemic strokes were recorded during the follow-up period. The stroke rate was not significantly different between patients with SR (3.4%) or AF (1.9%) at baseline (*p* = 0.46). In addition, a transient ischemic attack occurred in two patients, one in each group. There was no temporal relationship between any thromboembolic event and a documented episode of AF.

### Pharmacological antithrombotic treatment

The antithrombotic treatment prescribed after LAA closure varied substantially between patients and is reported in Table 3. At discharge, 93 patients (28.1%) received single antiplatelet therapy, 146 (44.1%) dual-antiplatelet therapy (aspirin and clopidogrel) and 80 (24.2%) short-term anticoagulation (alone or in combination with antiplatelet therapy). Only 12 (3.6%) patients remained without any antithrombotic agent. There was no significant difference in the antithrombotic treatment prescribed at discharge between AF and SR groups.

**Table 3:** Antithrombotic treatment at discharge

Patient characteristics	Sinus rhythm at admission n = 119	Atrial fibrillation at admission n = 212	*p*
			0.90
No antithrombotic agent	3 (2.5)	9 (4.2)	
Single antiplatelet therapy	34 (28.6)	59 (27.8)	
Dual-antiplatelet therapy	51 (42.9)	95 (44.8)	
Anticoagulation	19 (16.0)	32 (15.1)	
Anticoagulation + antiplatelet therapy	12 (10.1)	17 (8.0)	

### Antiarrhythmic treatment

Beta blockers were the antiarrhythmic agents the most commonly prescribed in this population (205 patients, 61.9%). Seventy-two (21.8%) and 28 (8.5%) patients received amiodarone or digoxin, respectively. Only 15 (4.5%) and 5 (1.5%) patients received class I or class IV (verapamil, diltiazem) antiarrhythmics. Most of the patients (215 patients, 65.0%) were treated with only one antiarrhythmic agent at discharge and 55 (16.6%) received two antiarrhythmic agents.

Among patients maintaining SR after one year follow-up, 90% were still under antiarrhythmic drugs.

## Discussion

### Major findings

The main findings of our study are: (1) procedures to restore or maintain SR, including external cardioversion or ablation, in this population, are rarely performed (only 4% of our population), with no major complication observed whatever the periprocedural antithrombotic protocol was. (2) the vast majority of patients undergoing percutaneous LAA closure will have the same AF status before and one year after the index procedure.

### AF evolution disease and LAA arrhythmogenicity

AF is commonly attributed to a trigger represented by pulmonary veins (PV) ectopies [17]. This location is particularly implicated in paroxysmal AF pathophysiology. In addition, atrial substrate abnormalities underlie AF maintenance. This substrate is usually located around PVs at the beginning of the disease but over time, the atrial substrate invades the left atrium and to a lesser extent the right atrium. This progression of the disease can be due to AF itself or other cardiac uncontrolled risk factors such as obesity, hypertension or diabetes and lead clinically to persistent or permanent AF. Atrial electrical foci location responsible for AF is a matter of debate as no clear consensus for their detection is available at the moment. LAA, even if rarely associated with AF trigger [18], have been identified in other previous studies, as a potential important origin for AF maintenance during persistent AF. Di Biase et al. [10] first, reported the potential arrythmogenic role of the LAA during AF. In this study, including 987 patients, LAA firing was observed in 27% of patients and was the only source of AF in 8.7% of this population. Other studies have also highlighted the role of LAA in AF maintenance. Notably, Lim et al. [19] demonstrated, using a 252-electrode vest for body surface mapping, that LAA could be a focal driver for persistent AF in 50–55% of cases.

Considering these reports, the LAA has been targeted during AF ablation. Di Biase et al. [20] demonstrated that LAA electrical isolation was associated with better outcomes for patients undergoing persistent AF ablation. Finally, a recent meta-analysis by Friedman et al. confirmed on 7 studies that LAA electrical isolation was associated with lower AF recurrences following AF ablation [21].

More recently, the potential arrhythmogenic effect of LAA closure on AF disease has come into question. Specifically, the LARIAT system, consisting in epicardial snared LAA occlusion (resulting in LAA ischemia) proved to increase significantly freedom from AF off antiarrhythmic drugs at 12 months follow-up (65% vs 39%) [12]. However, the arrhythmic effect of endocardial devices such as the Watchman or ACP/Amulet devices is unclear. Indeed, in contrary to surgical LAA snaring, percutaneous LAA closure could not alter the neuroendocrinal function of LAA. We have shown that, in our cohort of 331 patients undergoing endocardial LAA closure, 87% of those with SR at baseline were in SR at one-year follow-up. In addition, 93% of patients with AF at the time of LAA device implantation were still in AF during long term follow-up.

Numerous studies have evaluated the progression of AF disease over time. It has been conceptually established that AF disease progresses steadily from atrial ectopices to paroxysmal AF, persistent AF and finally permanent AF. The CARAF study evaluated 757 patients with paroxysmal AF and demonstrated that at one year follow-up, 8.6% of patients had developed chronic AF and 24.7% at 5 years. Age, significant aortic stenosis or mitral regurgitation, left atrial enlargement and underlying cardiomyopathy were independently associated with the evolution towards chronic AF [22]. More recently, De Vos et al. studied 1219 patients diagnosed with paroxysmal AF. After one year, 15% of these patients experienced AF disease progression [23].

Our results are consistent with the natural history of AF disease progression and suggest a neutral arrhythmogenic effect of endocardial LAA closure devices.

### AF management for patients with LAA occlusion

Sinus rhythm restoration is a cornerstone of AF therapy [14]. This can be achieved either using pharmacological or electrical cardioversion, or performing AF catheter ablation.

In Europe, percutaneous LAA closure is predominantly indicated for patients contraindicated to oral anticoagulation [14]. The FLAAC registry [15] evaluated prospectively the efficacy and safety on French patients undergoing percutaneous LAA closure from 2013 to 2015. Interestingly, the mean age of this population was 75 years and the mean CHA_2_DS_2_-VASc score was 4.5 indicating a population with numerous comorbidities. AF rate control strategies are usually preferred in this population rather than rhythm control strategies. This is consistent with the low rate of SR restoration attempts observed in our study (only 4% of the studied population), probably due to the potential complications related to electrophysiogical procedures in this frail population.

LAA closure has been proposed by some authors as a potential alternative to oral anticoagulation in patients without contraindication to OAC. In this specific population, rhythm control achieved with cardioversion or AF ablation is a common management strategy. Of note, patients undergoing rhythm control strategy in our population had a lower CHA_2_DS_2_-VASc (2.9 ± 0.4 vs. 4.6 ± 0.1, *p* < 0.001) and HAS-BLED scores (2.3 ± 0.3 vs. 3.2 ± 0.1, *p* < 0.01) than the general population included in the registry.

Moreover, as AF ablation requires LA catheterization, it has been proposed in small sample size studies to combine AF ablation and LAA closure [24, 25]. Gadiyaram et al. recently found that the Watchman or Lariat procedure for patients with electrical LAA isolation during AF ablation was safe and oral anticoagulation could be avoided in 98% of this population [26]. Li et al. [27] evaluated 25 patients undergoing combined AF ablation and LAA closure. In 24 patients, oral anticoagulant agents could be stopped at 6 months follow-up. All these studies include small sample sizes and larger series are required to support the efficacy and safety of this combined procedure.

Data evaluating the follow-up of patients with LAA closure undergoing subsequent cardioversion or AF ablation are sparse.

In our population, 7 patients underwent AF ablation. Five of these interventions were combined with LAA closure and two were performed 5 and 7 months after the index procedure, respectively. In addition, as shown in Table 4, one patient had no anticoagulation post-AF ablation without any thromboembolic event after the procedure.

**Table 4:** Rhythm control intervention during the follow-up period after LAA closure

Age (range)	Cardiac rhythm before LAA closure	History of thromboembolic event	History of AF ablation	CHA_2_DS_2_-VASc score	Procedure of interventional cardiology	Thomboembolic event after cardioversion or an ablation procedure	Antithrombotic treatment before the procedure	Antithrombotic treatment after the procedure
70′s–80′s	Sinus rhythm	No	No	2	Electrical cardioversion	No	VKA	VKA
80′s–90′s	Atrial fibrillation	Yes	No	5	Electrical cardioversion	No	Short term anticoagulation + Antiplatelet therapy	Low Molecular Weight Heparine + Antiplatelet therapy
80′s–90′s	Sinus rhythm	No	No	2	Overdrive pacing	No	Antiplatelet therapy	Antiplatelet therapy
70′s–80′s	Sinus rhythm	No	No	4	Overdrive pacing	No	None	None
70′s–80′s	Sinus rhythm	No	Yes	2	Atrial fibrillation ablation (CP with LAAC, Pulmonary *vein* isolation)	No	None	DOAC
60′s–70′s	Sinus rhythm	Yes	Yes	5	Left Atrial *flutter ablation (*Pulmonary *vein* isolation*)*	No	Antiplatelet therapy	Antiplatelet therapy
60′s–70′s	Atrial fibrillation	Yes	No	2	Right common *flutter ablation* (cavotricuspid isthmus *ablation)*	No	Antiplatelet therapy	Antiplatelet therapy
70′s–80′s	Sinus rhythm	No	No	4	2 ablation procedures: 1) CP with LAAC (cavotricuspid isthmus *ablation); 2) Subsequent ablation: (*Pulmonary *vein* isolation)	No	None VKA	Antiplatelet therapy None
50′s–60′s	Atrial fibrillation	Yes	No	3	Atrial fibrillation ablation (pulmonary *vein* isolation isolation) and right common *flutter ablation* (cavotricuspid isthmus ablation)	No	DOAC + Antiplatelet therapy	DOAC + Antiplatelet therapy
60′s–70′s	Sinus rhythm	Yes	Yes	4	Atrial fibrillation ablation (CP with LAAC, Pulmonary *vein* isolation)	No	DOAC	DOAC
60′s–70′s	Sinus rhythm	No	No	1	Atrial fibrillation ablation (CP with LAAC, Pulmonary *vein* isolation)	No	Antiplatelet therapy	Low Molecular Weight Heparine + Antiplatelet therapy
60′s–70′s	Sinus rhythm	No	No	2	Atrial fibrillation ablation (CP with LAAC, Pulmonary *vein* isolation)	No	Antiplatelet therapy + VKA	Antiplatelet therapy + VKA
60′s–70′s	Sinus rhythm	No	Yes	2	Atrial fibrillation ablation (CP with LAAC, Pulmonary *vein* isolation)	No	None	Low Molecular Weight Heparine + Antiplatelet therapy

CP with LAAC, Catheter ablation combined with left atrial appendage closure; DOAC, Direct Oral Anticoagulant; VKA, vitamin K antagonist

Electrical cardioversion can also be used to restore SR in persistent AF patients. This procedure is systematically followed by a minimum of 1 month of anticoagulation [14] but in some patients experiencing LAA closure, oral anticoagulants are strictly contraindicated and the need for cardioversion can occur in patients with LAA closure without anticoagulation. Electrical cardioversion following LAA closure have been described in some cohorts of patients. However, the anticoagulation protocol following cardioversion was not clearly described. In our prospective cohort, 4 patients experienced electrical cardioversion or overdrive pacing to restore SR. One patient had no anticoagulation before and after atrial arrhythmia reduction and had no thromboembolic event during the follow-up.

Cullen et al. [28] described 93 patients undergoing cardioversion after surgical LAA closure. They found a substantial number of patients (37%) with incomplete LAA closure and LA thrombus (28%) assessed on TEE, highlighting the need to exclude left atrial cavity thrombus before cardioversion in patients with LAA closure.

### Limitations

This study has several limitations. First, we included only patients with successful LAA closure and there was no control group. The AF recurrence rate after LAA closure was therefore compared to data reported in the literature. However, we believe that this indirect comparison provides interesting findings on the effect of LAA closure on cardiac rhythm. Secondly, most of the patients were old with a high rate of comorbidities and were mainly in persistent/permanent AF at baseline explaining that only of few of them underwent procedure to restore SR. Also this strategy could vary, depending on patient characteristics, individual preference or regional practices. Some of these cardioversions or ablation procedures were performed several months after LAA closure and the follow-up reported in this study for these patients was therefore limited. Our results on the safety of these procedures after LAA closure should therefore be taken with caution. Finally, AF status was only assessed according to ECG performed at baseline and at 1 year follow-up. Even if changes in AF burden assessed for instance by 24 h Holter-ECG, may be a more powerful outcome, our population represents a “real world” cohort of patients and we think that ECG modification between baseline and 1 year follow-up still represent an acceptable way to evaluate AF status modification.

## Conclusion

In our prospective cohort of 331 patients undergoing percutaneous LAA closure, AF status was not significantly influenced by percutaneous LAA closure after one year follow-up. Thirteen patients (4% of our population) underwent procedure to restore or maintain SR (cardioversion or ablation), some of them without oral anticoagulation. These patients were younger with lower comorbidities and none of them experienced thromboembolic event related to these procedures.

## Data Availability

All data generated or analysed during this study are included in this published article [and its supplementary information files]. The datasets used and/or analysed during the current study are available from the corresponding author on reasonable request.

## Abbreviations

AF: Atrial fibrillation
FLAAC Registry: French LAA Closure Registry
LAA: Left atrial appendage
OAC: Oral anticoagulants
SR: Sinus rhythm
TEE: Transesophageal echocardiography
